# 473. Clinical Outcomes of a Pediatric COVID Monoclonal Antibody Program based at an Urban Academic Center

**DOI:** 10.1093/ofid/ofab466.672

**Published:** 2021-12-04

**Authors:** Philip J Lee, Carlos Cruz, I J Anosike

**Affiliations:** Children’s Hospital at Montefiore, Long Island City, New York

## Abstract

**Background:**

Growing clinical evidence in adults has demonstrated use of COVID monoclonal antibody (mAB) therapy results in a reduction of hospitalization and/or emergency room (ER) visits with the greatest benefit following early administration. While the FDA has authorized use of mAB therapy in children ages 12-17 years, clinical outcomes in this population have yet to be described. This study aims to assess the pediatric clinical experience in a low social economic setting.

**Methods:**

Retrospective study conducted among children and adolescents who tested positive for SARS-CoV-2 from 12/1/2020 to 6/1/2021, met ≥ 1 eligibility criterion based on pre-determined institutional guidelines. Individuals were identified by patient-level data linked to pharmacy and medical claims with ICD-10 codes for COVID-19. Electronic medical records were reviewed for demographic characteristics, comorbidities, time to receipt of mAB therapy from positive test, adverse effects, and clinical outcomes. Primary clinical end point was hospitalization and/or medical visit at 28 days. Descriptive summary statistics were used for the entire cohort.

**Results:**

Overall, 17 met eligibility criteria. Thirteen patients with a mean age of 16 years, received casirivimab and imdevimab mAB therapy: 4 declined treatment. Among the treated patients, 61.5% (n=8) were male, 38.6% (n=5) Hispanic/Latino; 38.6% (n=5) non-Hispanic Black; 7.7% (n=1) White. Seven out of 12 had a BMI ≥ 95th% for gender and age. Eight patients (61.5%) met ≥ 1 criteria with obesity (n=8) as the most common factor followed by immunocompromised state (n=6, 46.2%) tied with neurodevelopmental disorder (n=6, 46.2%). Median time from positive test to mAB therapy was 2 days [IQR:1-3]. One patient had a severe adverse event. Overall, none required hospitalization/ER visit with COVID like symptoms.

Demographics and Results Table

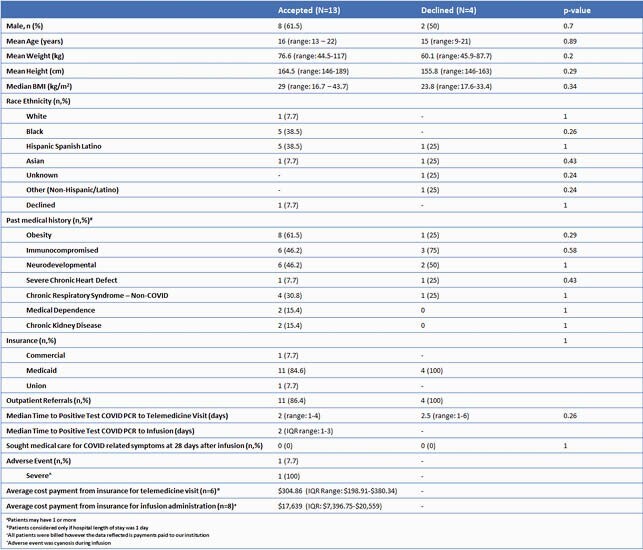

The table describes the pediatric patients and clinical outcome of receiving monoclonal antibody treatment for COVID-19.

**Conclusion:**

Though limited by numbers, our findings may suggest a role of mAB therapy in children and adolescents in our setting. With increasing rates of SARS-CoV-2 in this age group coupled with vaccine hesitancy, mAB therapy may serve as an important outpatient intervention with a need for further studies to assess clinical benefit and establish optimal, cost-effective, practice guidelines for these highly vulnerable patients.

**Disclosures:**

**All Authors**: No reported disclosures

